# Supplementary material for: Are carbon emissions trading and green financial instruments synergistic? -Comprehensive quantitative research based on content analysis
Source: PLoS One. 2024 Mar 7;19(3):e0298601. doi: 10.1371/journal.pone.0298601 (PMC10919601; doi:10.1371/journal.pone.0298601)
Supplement: S1 Data — (ZIP) [file pone.0298601.s001.zip › Data/地级市Z_F工作报告.zip!/╔╜╢1⁄2/╢1⁄2╙¬╩╨/└┤╫╘╣1⁄2╓┌║┼í╛┬φ┐╦╩2╛▌═°í┐_2013.docx]

各位代表：

　　现在，我代表市人民政府向大会报告政府工作，请予审议，并请市政协委员和其他列席会议的同志提出意见。

　　一、2012年工作回顾

　　过去的一年，是深入贯彻市第五次党代会精神、全面建设生态文明典范城市的开局之年，也是我们迎难奋进、成果丰硕的一年。一年来，在省委、省政府和市委的正确领导下，全市上下以科学发展观为指导，紧紧围绕实施黄蓝国家战略、建设生态文明典范城市，凝心聚力，锐意进取，扎实工作，经济社会发展取得显著成绩。

　　(一)经济保持平稳较快发展。在外需严重萎缩、经济下行压力加大的情况下，我们加强形势研判，及时出台措施，经济发展逆势而上，实现新的突破。初步统计，2012年全市生产总值突破3000亿元大关，规模以上工业产值突破万亿元大关，主营业务收入过百亿元企业达到21家，固定资产投资、进出口、公共财政预算收入、服务业、金融机构贷款增幅均居全省前列。大力实施项目带动战略，开工建设投资过亿元项目450个，其中过10亿元项目62个，分别增长25.7%和44%，全社会固定资产投资达到1900亿元，增长23.2%。全力保障企业健康运行，落实减轻企业负担、改善企业资金供应等政策措施，工业发展质量、效益同步提高，增加值、利税分别增长12.5%、12.9%。持续扩大消费需求，认真落实鼓励消费政策，努力保持市场物价稳定，社会消费品零售总额达到516亿元，增长15%。强化财税金融支撑，公共财政预算收入158.71亿元，增长17.33%；金融机构各项贷款余额1806亿元，比年初增加367.2亿元。

　　(二)产业结构调整步伐加快。抓住我市整建制列为国家现代农业示范区的机遇，大力发展高效生态农业。绿色种植、生态养殖规模效益不断扩大，建设粮食高产创建示范片10万亩，海参、大闸蟹等特色水产品养殖达到187万亩，标准化畜牧养殖小区和万头奶牛养殖场分别达到930个、3个，工厂化食用菌生产能力达到30万吨，“三品”生产基地达到160万亩。农业产业化水平不断提升，农业龙头企业达到560家，农民合作社发展到765个。农业园区蓬勃发展，东营农业高新技术产业示范区基础设施和项目建设全面展开，被列为国家农业科技园区。加强农业基础设施建设，完成灌区改造、河道治理等一批农田水利工程，改造中低产田6.7万亩。强农惠农富农政策全面落实，市级财政“三农”支出达到11.7亿元，增长16.73%。推进工业优化升级。改造提升石油化工、橡胶轮胎等优势产业，实施了东营联合石化、胜利石化、中海石油东营石化3个500万吨炼化和渤海湾1500万吨原油上岸终端等项目。发展壮大石油装备、汽车及零部件等先进制造业，出台加快石油装备产业发展的政策措施，实施了一批大项目。积极培育电子信息、新能源等战略性新兴产业，全市高新技术产业比重达到32.5%，比年初提高1.6个百分点。加快发展现代服务业。制定出台推进服务业跨越发展若干政策，大力培植“四大载体”，服务业增加值增长12.5%。文化旅游业快速发展，龙悦湖旅游度假区一期工程建成投用，黄河口生态旅游区游客服务中心、鸟类救护科普中心主体完工，孙子文化旅游区成为国家4A级景区，黄河三角洲动物乐园投入运营。金融业加快发展，设立了石油装备产业股权投资基金，黄河三角洲产权交易中心挂牌运营，银行业金融机构达到21家。现代物流、服务外包、会展等产业发展加快。着力提高科技创新支撑能力。黄河三角洲可持续发展研究院、石油大学科技园等创新平台建设深入推进，山东大学东营研究院挂牌运行，国家采油装备工程技术研究中心通过可行性论证，新建省级以上企业技术中心、院士工作站10家。加强与高等院校和科研机构合作，与5所大学签署了合作协议。我市被认定为首批国家知识产权示范城市，获准创建首批全国质量强市示范城市。

　　(三)生态环境建设成效显著。坚持把生态环境建设放在突出位置，全面构筑生态系统，实现突破性进展，成为生态文明典范城市建设的新亮点。大力推进造林绿化。11处林场全面完成年度建设任务，造林15.6万亩，探索出了造林绿化新模式。生态绿化工程深入实施，植树800多万株。环城生态绿化、东青高速公路绿化、广利河绿化等工程开工建设。加快构筑循环水系。全面启动金湖银河生态工程，开挖生态河道31.4公里，引黄河水自流入城；实施东城水系内循环工程，贯通27平方公里城市水系，水城景观进一步显现。加强湿地修复，自然保护区湿地修复和淡水补给工程全面完工，实现补水35万亩，东八路湿地建设顺利推进。全面实施公共环境改善工程，水气污染和城市扬尘治理取得显著成效。实施企业污染治理工程610个，小清河等流域生态治理工程顺利推进，重点河流水质稳定好转。新建改造西城南等污水处理厂20座、垃圾处理场1座，生活垃圾焚烧发电厂一期工程竣工，中心城工业企业搬迁改造扎实推进。国家环保模范城市持续改进工作通过复核。荣获“中国温泉之城”称号。生态文明乡村建设深入开展，城乡环卫一体化基本实现。强力推进节能减排。积极推进省级低碳生态示范城市建设，加强行业准入和源头控制，加大重点领域、行业和企业监管力度，淘汰落后产能，加快发展循环经济，万元生产总值能耗等均可实现控制目标。

　　(四)城市和基础设施建设实现重大突破。实施了总投资181亿元的两批57个重点城建项目，拉开中心城跨越发展新格局。加快城市绿化美化，实施拆墙透绿、见空插绿，建设森林公园、城市公园、街心公园45处，新增绿地400万平方米，改造提升东二路、西四路等28条道路74公里，完成东三路、辽河路等7条道路沿街立面改造84万平方米，城市面貌焕然一新。加快公共设施建设，城市规划展览馆、黄河文化博物馆基本完工，大剧院、科技馆、图书馆、青少年宫、奥体中心完成年度计划。生态新城开工建设，东城三大片区和西城改造扎实推进。城市管理体制进一步理顺，长效管理机制初步建立。“五城联动”环境整治取得明显成效，国家卫生城市通过复审。积极推进县城和小城镇建设，新建改造新型农村社区20个、农村公路500公里，建设农村住房1.2万户，投资4亿元实施农村电网改造。加快推进重大基础设施建设。东营港十大业主码头和公共管廊带建设基本完工，南港池改造进展顺利。胜利机场配套设施不断完善。飞机场至自然保护区公路建成通车，德大铁路建设加快推进，济东高速公路、寿平铁路开工建设，广利港前期工作进展顺利。东营港防潮堤陆上段全面竣工，海上段顺利合龙。南水北调工程顺利实施。胜利电厂三期工程开工建设。

　　(五)改革开放扎实推进。深化重点领域和关键环节改革。未利用地开发取得突破，在全省率先实施了7个开发项目，已开发完成2.9万亩，净增耕地1.3万亩。农村综合改革扎实推进，完成农村集体土地所有权登记颁证工作，农村土地承包经营权流转有序开展，集体林权制度配套改革进展顺利。行政审批制度改革深入推进，取消、下放和调整行政审批事项70项，取消行政事业性收费12项。事业单位分类改革稳步推进。积极扩大对外开放。出台促进对外贸易稳定增长的意见，成功举办国际石油石化装备展、橡胶轮胎展、农博会等大型经贸活动，加大重点出口基地和骨干企业扶持力度，进出口总值达到122亿美元，其中出口49亿美元，分别增长19%和12.4%。出台进一步做好利用外资工作的意见，加强招商选资，与央企和世界500强战略合作深入推进，实际利用外资1.6亿美元，增长14%。加快企业“走出去”步伐，境外投资增长18%，对外工程营业额4.2亿美元。加强对外交流合作，与美国麦迪森威尔市、澳大利亚天鹅市建立友好交往城市关系，与韩国群山市建立经济合作伙伴关系。

　　(六)区域经济协调发展。胜利油田持续稳产高产，在勘探开发、生产经营、市场开拓、内部改革管理等方面取得新成效，实现了又好又快发展。石油大学在教学科研、科技合作等方面取得突出成绩，实现了重大进展。济军生产基地努力培育新的经济增长点，实现了新的发展。加快发展市属经济，东营经济技术开发区实施了66个过亿元项目，高端产业加快聚集；东营港经济开发区实施了60个重大产业项目，发展速度明显加快。加大促强扶弱力度，出台支持利津加快科学发展、加快试点强镇建设等政策意见，县域经济实力进一步增强。

　　(七)保障改善民生水平明显提高。全面落实民生政策，大幅增加民生投入。2012年，市级财政民生支出比重达到42.26%，提高2.54个百分点。十大民生工程完成年度计划。积极推进创业就业，制定鼓励开展创业富民若干政策，广泛开展新型农民学校创建，培训农民21.6万人次，新增城镇就业4.97万人，农村劳动力转移就业4.1万人，城镇登记失业率控制在2%以内，城镇居民人均可支配收入31035元、增长13.5%，农民人均纯收入11580元、增长15.5%。健全完善社会保障体系，城乡居民基础养老金由每人每月60元提高到120元，比国家规定标准高1倍多；城镇居民医保、新农合财政补助标准由每人每年210元提高到260元，在全省率先启动了整合试点工作；城市低保标准提高到每人每月430元以上，农村低保、五保供养标准分别提高到每人每年2660元、5000元；企业退休人员月人均养老金提高到1747元，企业最低工资标准提高到每月1240元，社会保障水平位居全省前列。加快实施城乡安居工程，建设保障性住房1.4万套，超额完成省下达的任务。支持油田建设职工住房4624套。对中心城7处农贸市场实施改造，市场环境明显改善。

(八)各项社会事业全面进步。加大教育投入，实施了中小学办学条件标准化、中小学校舍安全、幼儿园办园条件标准化、中心城区义务教育学校、东营职业学院“三中心”、市技师学院二期等工程项目，为全市在园孤儿、残疾幼儿、家庭经济困难幼儿发放保育费补助，为农村义务教育阶段学生发放营养餐补助，为义务教育阶段低保家庭学生免费提供学生装，为城市义务教育在校生免除课本费、作业本费。大力发展卫生事业，市人民医院急诊急救中心、市立儿童医院开工建设，市疾病预防控制中心检验综合楼主体完工；医药卫生体制改革深入推进，公立医院改革全面推开，9家公立医院实行药品零差价销售，群众“看病难、看病贵”问题得到有效缓解。繁荣发展文化事业，公共文化设施建设加快推进，成功举办孙子国际文化旅游节、黄河口文化艺术节等活动，群众文化生活日益丰富。加强精神文明和民主法制建设，“六五”普法工作扎实开展。全面启动创建全国文明城市工作，严格落实网格监管责任，加大市容环境、居民小区、交通秩序、市场秩序等整治力度，广泛开展义务劳动和志愿者服务，提升市民思想道德素质和社会文明程度，创城工作取得第三名的好成绩。积极开展全民健身运动，成功举办了国际马拉松赛、市九运会等大型赛事，竞技体育取得优异成绩。低生育水平持续稳定，出生人口素质不断提升，“幸福家庭”创建活动深入推进，人口计生工作成效显著。积极推进社会管理创新，扎实开展信访工作，加强社会管理综合治理，社会治安监控系统建设任务圆满完成。高度重视安全生产，启动创建国家安全发展示范城市工作，扎实开展安全生产基层基础强化年活动，安全生产形势持续稳定。加强食品药品安全监管，建立健全食品安全监管体制机制，保障人民群众饮食用药安全。国防教育和双拥共建深入开展，军政军民团结更加巩固。对口帮扶新疆疏勒取得新成效。新闻出版、外事侨务、防震减灾、人民防空、民族宗教、社会科学、妇女儿童、老龄、青少年、残疾人、慈善和红十字会等事业健康发展，应急管理、对台、统计、档案、史志、仲裁、气象、无线电管理等工作都取得新成绩。

　　各位代表，建设生态文明典范城市离不开外力的支持。一年来，我们紧紧抓住实施黄蓝国家战略重大机遇，积极争取上级支持，取得一系列突破。政策方面，国家同意我市开展低丘缓坡荒滩未利用地开发利用试点；初步确定东营为国家资源型城市可持续发展试点城市；东营经济技术开发区被列为国家循环化改造示范试点园区，省政府批准设立副厅级管理机构。项目方面，东营港防波堤建设纳入交通部“十二五”规划；滨海新城防潮堤、黄河下游防洪工程、大唐东营电厂、500千伏黄河输变电工程获得国家支持；中国商飞公司确定在胜利机场建设民用飞机试飞基地。资金方面，全年争取上级扶持资金46.6亿元。这些政策、项目、资金的注入，为东营推进科学发展、跨越崛起增添了强大动力。

　　各位代表，过去一年的成绩来之不易，这是省委、省政府和市委正确领导的结果，是市人大、市政协和社会各界有效监督、大力支持的结果，是全市上下、油地军校同心同德、团结奋斗的结果。在此，我代表市人民政府，向辛勤工作在各条战线上的广大干部群众，向各民主党派、工商联、各人民团体和各界人士，向驻东营人民解放军、武警官兵和中央、省驻东营各单位，向所有关心、支持东营发展的海内外朋友，致以崇高的敬意，表示衷心的感谢！

　　二、面临的形势和2013年工作总体要求

　　2013年，是全面贯彻落实党的十八大精神的开局之年，是全力实施黄蓝国家战略、实现“两个率先”进程中的重要一年，我们也将迎来建市30周年。做好今年的政府工作，意义重大而深远。

　　目前，国内外形势十分严峻。国际经济形势依然错综复杂、充满变数，世界经济低速增长态势还将延续。我国发展面临不少风险和挑战，不平衡、不协调、不可持续问题依然突出，经济增长下行压力仍然很大。我市发展也面临许多矛盾和问题。受经济大环境影响，企业生产经营形势不容乐观，特别是中小企业在资金融集、市场开拓等方面存在较多困难；经济结构不合理，产业层次较低，自主创新能力不强，转型升级任务艰巨；资源环境约束趋紧，节能减排压力巨大，生态环境建设任务繁重；防御自然灾害能力不够强；改革开放有待深化，体制机制障碍仍然存在，对外开放程度不高，利用外资规模较小；社会管理创新亟待加强，一些关系群众切身利益的问题还没得到很好解决。

　　各位代表，形势越是复杂严峻，我们越要把握有利条件，坚定发展信心。党的十八大胜利召开，描绘了全面建成小康社会、加快推进社会主义现代化的宏伟蓝图，为我们今后的发展指明了方向。市委五届二次全会提出的“两个率先”的奋斗目标，完全符合党的十八大精神，符合东营实际，落实好、实现好这一奋斗目标，必将引领东营走向繁荣富强。随着黄蓝国家战略深入实施，东营的知名度越来越高，影响力越来越大，为我们创造了更加广阔的发展空间。经过三十年的发展，我市综合实力大幅提升，内生动力明显增强，特别在近几年应对国际金融危机过程中，我市经济经受了检验和砺炼，企业竞争力不断提升，产业规模不断扩大，发展支撑条件更加稳固。我们务必准确把握形势，切实增强机遇意识、忧患意识，充分利用有利条件，努力克服不利因素，牢牢把握工作主动权。

　　今年政府工作的总体要求是：深入学习和全面贯彻落实党的十八大精神，以邓小平理论、“三个代表”重要思想、科学发展观为指导，紧紧围绕主题主线，以黄蓝国家战略统领发展全局，加快调整经济结构，全面推进生态建设，不断深化改革开放，着力保障改善民生，实现经济持续健康发展和社会和谐稳定，为率先全面建成小康社会、率先建成生态文明典范城市打下坚实基础。

　　今年全市经济社会发展的预期目标是：生产总值增长12%，全社会固定资产投资增长20%，社会消费品零售总额增长15%，进出口总值增长12%，吸收外商直接投资增长10%，公共财政预算收入增长14%，城镇居民人均可支配收入增长12%，农民人均纯收入增长13%，城镇登记失业率控制在2.4%以内，居民消费价格涨幅控制在3.5%左右，人口自然增长率控制在4.74‰以内，全面完成省政府下达的各项节能减排约束性目标。

　　面对当前形势，实现今年发展目标，必须牢牢把握全面贯彻落实党的十八大精神这一核心，深刻领会精神实质，将其贯穿于经济社会发展全过程，落实到政府工作各环节，奋力开创科学发展新局面。必须牢牢把握实现“两个率先”这一总目标，充分发挥实施黄蓝国家战略、自然资源丰富、经济综合实力较强等优势条件，调动方方面面积极性主动性创造性，着力解决城乡发展、城市内部发展、经济与社会发展三个不平衡问题，努力实现更加协调更高质量的发展，推动东营各项事业继续走在前列。必须牢牢把握产业结构调整这一关键，大力发展高效生态农业、环境友好型工业和现代服务业，做大做强优势产业，培育壮大新兴产业，加快构筑现代产业体系。必须牢牢把握改革开放创新这一强大动力，深化重点领域和关键环节改革，更加积极主动地推进对外开放，实施创新驱动发展战略，激发经济社会发展新活力，增创体制机制新优势。必须牢牢把握保障改善民生这一根本，努力增加城乡居民收入，在教育、就业、社会保障、医疗、住房、生态环境、社会治安等方面，多为群众办实事、办好事，不断增进人民群众福祉。必须牢牢把握项目建设这一抓手，把全年的工作任务细化为具体项目，实行项目化管理、项目化运作，全力攻坚突破，以大项目带动大投入、推动大开发大发展。只要我们全市上下、油地军校、社会各界同心同德，奋力拼搏，创新进取，就一定能攻坚克难，乘势而上，夺取经济社会发展的新胜利！

　　三、2013年重点工作

　　(一)着力应对宏观环境变化，在保持经济持续健康发展上取得新成效。保持经济持续健康发展，是今年经济工作的首要任务。必须准确把握经济走势，认真落实国家宏观调控政策，加强经济运行分析调度，及时采取应对措施，继续保持良好发展势头。

　　保持投资稳定增长。在当前情况下，投资仍然是经济增长的重要拉动力。要坚持投速、投量、投向、投效有机统一，保持投资合理增长。以项目建设带动投资，加快推进284个亿元以上续建项目，实施好268个亿元以上新开工项目。着力优化投资结构，引导各类资金投向现代农业、高新技术、技术改造、现代服务业、社会民生等重点领域。筛选储备一批对结构调整和产业升级具有引领作用的大项目、好项目，搞好项目接续。落实政策措施，面向全社会推出一批民间投资参与的具体项目，促进民间投资稳定增长。

　　积极扩大消费需求。深入实施市民增收工程，按照收入倍增要求，落实创业富民政策，千方百计增加城乡居民特别是低收入群众收入。认真落实各项鼓励消费政策，积极培育社会养老、家政服务、体育健身、文化娱乐、节能环保等新的消费热点。深入实施“万村千乡”市场工程，支持供销系统建设农产品现代流通网络，基本完成中心城农贸市场改造，抓好摩托车下乡和建材下乡试点。大力推进电子商务、连锁经营和物流配送，加快培育电子商务平台。着力优化消费环境，维护消费者权益。加强调控监管，保持物价总水平基本稳定。

　　保障企业健康发展。企业是经济之源、发展之本。必须牢固树立为企业服务的理念，继续强化扶持措施，为企业发展创造良好环境。抓融资服务，完善政银企合作机制，引导金融机构扩大信贷投放，提高中小微企业信贷资金比重。抓市场服务，指导企业加强品牌建设，创新营销手段，扩大市场占有率。抓技术服务，支持企业瞄准高端延伸产业链条，提升研发能力和装备水平，促进产品升级换代。抓管理服务，引导企业加强管理创新，规范运作，降本增效。抓政务服务，落实企业减负、税收优惠等政策，全面清理和规范各种收费，打造一流的政务环境。

　　强化财税金融支撑。加强高效财源建设，加大依法治税管费力度，保持财政收入稳定增长，提高财政收入质量。优化财政支出结构，集中财力保民生、保重点、保在建。继续深化财政改革，推进预算决算公开。强化审计监督，维护良好的财经秩序。大力推进金融创新，培育壮大产业投资基金，搞好黄河三角洲产权交易中心运营。支持金融机构健康发展，稳步发展农村商业银行、村镇银行和小额贷款公司。搞好区域集优债务融资试点，发展金融机构表外融资业务，鼓励企业通过上市、发行债券扩大直接融资规模，降低实体经济发展的融资成本。强化金融风险防范和监管，抓好民间融资规范引导试点。

　　(二)着力调整优化经济结构，在加快转型发展上取得新成效。调整优化经济结构、加快产业转型升级，是东营的一项紧迫任务。我们必须坚定不移推进经济结构战略性调整，提升产业竞争力，提高经济增长的质量和效益。

　　推动工业由大变强。一是积极培育战略性新兴产业。围绕新能源、新材料、新医药、新信息和海洋开发等重点领域，加强规划引导，重点抓好66个过亿元项目，推动战略性新兴产业发展成为支柱产业。二是发展壮大先进制造业。汽车及零部件产业，大力培植龙头企业，加强协作配套，重点抓好14个过亿元项目，尽快形成规模优势。石油装备产业，以东营经济技术开发区和胜利经济开发区两大集聚区为重点，加快推动资源整合、要素聚集、科技创新，重点抓好25个过亿元项目，搞好国家级石油装备质检中心建设，不断提升石油装备产业在全国的主导地位。三是加快改造提升传统优势产业。坚持高新化与集群化发展方向，运用高新技术嫁接改造传统产业，促进产业向高端提升、产品向高端发展；推动产业向园区聚集，实现集约集聚发展。石化产业，坚持“压小上大”，加快改造整合，重点抓好3个大型炼化项目，形成从油头到化尾的循环产业链。橡胶轮胎产业，加强技术研发、品牌培育和流通体系建设，促进骨干企业做大做强。四是推进信息化和工业化深度融合。抓好7个重点项目建设，支持企业运用信息技术提升自动化、智能化、管理现代化水平。

　　推动服务业跨越发展。一是突出发展生产性服务业。加快企业主辅分离，完善政策措施，扶持现代物流、信息服务等产业发展。大力发展现代物流业，重点依托东营港、广利港、飞机场、火车站建设物流园区，加快中海油物流园等项目建设，培育一批大型物流企业。积极发展服务外包业，推动软件园区和服务外包企业提高层次、扩大规模。二是积极发展生活性服务业。在城市，重点发展文化娱乐、社区服务等行业。在农村，结合新型农村社区建设，推动超市、托幼、养老等服务网点健康发展。加快发展文化旅游业，突出抓好黄河口生态旅游区、龙悦湖旅游度假区、孙子文化旅游区、吕剧文化产业园等园区建设，新上一批特色精品项目，打造“黄河入海·龙腾东营”旅游品牌。三是统筹发展各类服务业。改造提升批发零售、商贸餐饮等传统服务业，加快发展商务服务、会展、休闲健身、家庭服务等新兴服务业，促进房地产业健康发展。

　　推动区域经济协调发展。培育壮大三个增长极，加快发展市属经济。中心城区，加快实施23个重点服务业项目，促进现代服务业聚集发展。东营经济技术开发区，突出发展汽车及零部件、电子信息、新能源等高端产业，重点实施20个大项目，加快打造高端产业聚集区。东营港经济开发区，按照港区城一体的思路，大力发展临港经济，实施26个大项目，加快建设现代生态化工基地。大力发展县域经济，坚持放权放活，支持各县区提升标杆，跨越发展，打造各具特色的经济隆起带；加大促强扶弱力度，促进强县强镇率先发展，全力支持利津加快科学发展。高举油地军校团结发展旗帜，紧紧围绕实现“两个率先”目标，凝聚各方力量，共享区域资源，共创发展优势，共促社会和谐，共建美好家园。

　　(三)着力发展现代农业，在解决“三农”问题上取得新成效。发展现代农业、促进农民持续增收，是解决“三农”问题的关键。要以建设国家现代农业示范区为总抓手，创新农业经营体制机制，加快构建现代农业产业体系，壮大农村经济，促进农民收入持续较快增长。

　　加快农业园区发展。坚持规划引领、政策资金支持、大项目支撑，市和县区分别抓好一批农业重点园区，推动东营农业高新技术产业示范区创建国家农高区，进一步提升国家现代农业示范区建设层次和水平。加快推进40个过亿元项目，带动农业结构优化升级。以提质增效为中心，以发展高效特色农业和生态循环农业为重点，围绕渔业、畜牧、林果、蔬菜、花卉和休闲观光农业六大主导产业，建设一批高标准生产基地。开展粮棉菜高产创建活动，提高主要农产品供给保障能力。

　　进一步提升农业产业化水平。培育发展农业龙头企业，对农民增收带动作用突出的龙头企业给予重点扶持，力争年销售收入过亿元龙头企业发展到90家。加大黄河口特优农产品品牌培育和宣传推介力度，健全完善营销网络，积极推动产销对接。加快发展各类农民合作社，力争新建100家，带动更多的农户进入产业链经营。高度重视农产品质量安全，继续实施放心蔬菜惠民工程。

　　切实加强农业农村基础设施建设。突出抓好农田水利工程建设，加快推进南水北调配套工程、灌区节水改造工程，疏浚治理6条河道，建设5座引黄泵站和2座水库，建设高标准农田、改造中低产田7.2万亩。新建改造农村公路400公里，搞好农村电网续建配套。深入实施农村饮水安全工程，解决2.2万人的饮水安全问题。促进农机与农艺结合，提升农业机械化水平。

　　不断拓宽农民增收渠道。全面落实各项强农惠农富农政策，增加农民转移性收入。大力发展农村二、三产业，不断壮大农村集体经济，支持农民自主创业，增加农民工资性收入。完善农村产权制度和征地制度，积极稳妥地促进土地承包经营权流转，增加农民财产性收入。办好中德农牧业技术培训中心，加强对基层技术人员和生产大户的科技培训，增强农民增收致富能力。

　　(四)着力推进新型城镇化，在打造秀美宜居城市上取得新成效。新型城镇化是扩大内需的最大潜力，是推动经济发展的巨大引擎。我们要按照“一主、四次、二十个小城镇、二百个新型农村社区”四级新型城镇体系的布局，以“提质加速、城乡一体”为目标，以“人的城镇化”为核心，积极稳妥推进新型城镇化发展。

　　增强区域中心城市辐射带动作用。以东城、西城、垦利县城和滨海新城为市域功能组织核心，进一步优化空间结构和产业布局，实施生态对接，促进组团发展，提高中心城辐射带动能力。加强重大城建项目建设。加快推进城市规划展览馆、科技馆、青少年宫等重大工程，确保按期建成投用。新开工一批重大项目，城市道路重点抓好庐山路等道路建设，景观提升重点抓好植物园景观提升等工程，片区开发重点抓好生态新城、金湖银河等片区，西城改造重点抓好黄河路两侧改造等工程，市政公用设施重点抓好生活垃圾压缩转运站、东一路供热站增容等项目。强化县域中心城市在城镇化中的骨干支撑作用，积极推进河口城区、广饶县城、利津县城和东营港城建设。

　　积极推进小城镇和新型农村社区建设。加强小城镇规划建设，进一步提升吸引力、带动力和综合实力。推进市级试点强镇、省级示范镇和国家级改革试点镇加快发展，利用三年时间，将其建成县域经济社会发展次中心。抓好生态文明和谐油区乡镇创建工作。有序推进新型农村社区建设。坚持以人口向社区集中、土地向规模经营集中为导向，科学合理推进迁村并点和合村并居。因地制宜确定规划设计风格和建设模式，突出农村社区特色，方便群众生产生活。加快推进城中村、城边村、空心村、乡镇驻地村拆迁改造，搞好规划、精心组织，实施好黄河展区村搬迁改造工程，力争三年基本完成任务。强化城镇公共服务功能。加强城镇市政设施建设，完善提升科技、教育、文化、体育、医疗、养老等服务设施，推动公共服务向基层和社区延伸。深化户籍、土地管理制度改革，有序推进农业转移人口市民化。

　　切实加强城市管理。推进“智慧城市”建设，完善城市地理信息、交通通信、社会治安等智能化信息系统，提高城市信息化水平。推行“数字城管”和网格化管理，实施综合执法，构筑“大城管”格局；推动城市管理向镇村延伸，建立小城镇综合执法、绿化养管、环卫保洁队伍，提高城镇管理水平。加强社区物业管理，理顺管理体制，规范物业收费，完善基础设施，建设一批示范社区、示范物业公司。深化环境综合整治，突出抓好老旧小区、背街小巷等薄弱环节，提升城市整体形象。努力争创中国人居环境奖。

　　着力加强重大基础设施建设。东营港，建成投用公共管廊带和十大业主码头，加快内港池整治和南港池开发，力争年内实现东营至旅顺滚装船航线复航，启动建设一批新码头。完善提升广利港规划，尽快开工建设。增强胜利机场服务功能，发展民航产业，建设民用飞机试飞基地，进一步增开航线航班。加快德大铁路、济东高速公路建设进度，规划建设东营火车站。加快推进黄河下游防洪工程建设。完善沿海防潮体系，年内完成东营港防潮堤工程，力争开工建设滨海新城防潮堤和北部沿海防潮堤。加强能源建设，建设一批风力发电和光伏发电项目，实施一批输变电工程。抓好新型信息技术类的基础设施建设。

　　(五)着力完善生态系统，在改善生态环境上取得新成效。党的十八大把生态文明建设提到了“五位一体”总布局的高度，我们要把生态作为建设生态文明典范城市的基础工程核心工程，一以贯之抓下去，全面构筑生态系统，加快建设生态东营。

　　加强生态建设。以创建国家生态园林城市为抓手，加快造林绿化，基本完成11处林场建设任务，探索完善长效经营管理机制，不断提高建设管理水平；实施沿黄、沿海生态工程，构筑城市绿色生态屏障；大力提高城市绿化水平，加快推进环城生态工程，年内完成东青高速公路绿化、广利河沿河绿道和滨河绿带建设，实施一批城区道路绿化和公园建设工程。做好山东省第五届城市园林绿化博览会前期筹备工作和园博园建设。继续实施金湖银河生态工程，加快推进东湖、西湖、中央湖景观和生态河道建设，形成生态水系大循环。加强湿地生态保护，搞好自然保护区10万亩湿地修复，加快东八路湿地绿化改造和配套设施完善，打造“东方湿地之城”。

　　狠抓节能减排。强化源头控制，严格执行能评、环评制度，严禁新上高耗能、高排放项目，坚决淘汰落后产能。突出抓好重点行业、重点企业监管，落实监测预警、合同能源管理等制度。积极推广新技术、新产品，实施10项节能减排工程，提升企业技术装备水平。大力发展循环经济，推行清洁生产，完善石油化工、造纸等10个循环经济产业链，培育一批循环经济园区。推进建筑、交通、公共机构和居民生活等领域的节能，新购置一批环保燃气公交车。全面落实最严格的水资源管理制度，建设节水型社会。认真落实土地利用总体规划，依法集约高效利用土地。科学开发利用地热等矿产资源。

　　强化环境整治。深入实施公共环境改善工程。全面加强省控、市控重点河流整治，确保水质稳定达标。加快实施新一轮小清河流域生态综合治理。尽快解决城市建成区污水直排问题。强化大气污染治理，严控工业废气、城市扬尘、机动车尾气污染。严格环境执法，严肃查处偷排偷放、超标排放等违法行为，依法取缔土小污染企业。集中整治破坏生态环境和危害群众健康的突出环境问题。完善环境基础设施，加快推进西城北、东城南等5座污水处理厂和生活垃圾焚烧发电二期等项目建设，实施好3座污水处理厂提标工程，建设3处环卫工作站。深入开展生态文明乡村建设，加强农业面源污染治理，搞好村容村貌综合整治，改善农村生态环境。

　　(六)着力推进改革开放创新，在增强发展动力活力上取得新成效。改革开放创新是实现“两个率先”目标的强大动力，必须以更大气魄、更大力度、更宽胸怀推进改革开放，加快创新驱动步伐，以改革开放创新的新成效推动经济社会科学发展、率先发展。

　　全面推进各项改革。深化投资体制改革，规范政府投融资平台管理，放大资本效用，提升运营能力。启动低丘缓坡荒滩未利用地开发利用试点，加快推进4个未利用地开发项目；推进城乡建设用地增减挂钩试点。稳步推进农村综合改革，扎实做好各类农村产权的确权登记和颁证工作，继续深化集体林权制度配套改革，加强农村集体资源、资产、资金管理，探索建立发展村级公益事业的新机制。深化行政管理体制改革，加快转变政府职能，稳步推进事业单位分类改革；扩大县区和开发区权限，扎实推进经济发达镇行政管理体制改革试点，搞好6个试点强镇和4个省级示范镇建设。加快推进公共资源交易管理体制改革。积极探索建立资源节约和环境保护机制、资源与生态补偿机制，推行排污权交易等市场化运作模式。积极推进水权制度改革。

　　进一步扩大对外开放。大力实施招商引资、招商选资，加强与央企、世界500强战略合作，吸引跨国公司地区总部和功能性机构落户我市，扩大外资规模，提升外资质量。实施市场多元化战略，巩固扩大发达经济体市场份额，深度拓展新兴市场，提高自贸区市场占有率，推动出口持续增长。加快转变外贸增长方式，重点支持自主品牌和高附加值产品出口，促进加工贸易转型升级。帮助企业用好国家鼓励出口政策，积极应对贸易摩擦。办好国际石油石化装备展等大型展会，积极申报国家级综合保税区。扩大先进技术设备、关键零部件和能源原材料大宗商品进口。推动电子口岸建设，打造便捷高效的大通关体系。加快“走出去”步伐，鼓励有条件的企业到境外建立生产基地、合作开发资源、承揽工程。

　　实施创新驱动发展战略。认真落实鼓励创新的政策措施，注重发挥企业家才能，加快科技创新、品牌创新、产业组织创新、商业模式创新。加大科技投入，全社会研发投入占生产总值比重提高0.1个百分点，重点企业科技经费支出占销售收入比重达到5%以上。建设一批科技创新平台，加快建设黄河三角洲可持续发展研究院、山东大学东营研究院，积极引进高层次研发机构；依托重点企业、科研院所，组建国家和省级工程技术研究中心、重点实验室、企业技术中心。建立一批技术创新联盟，在优势产业和战略性新兴产业等领域加强协同创新。发展一批创新型企业，突出企业在技术决策、研发投入、科研组织和成果转化中的主体地位，增强企业核心竞争力；推动科技与金融结合，积极搭建平台，支持科技型中小企业发展。实施一批重大科技专项，着力解决制约产业发展的关键技术难题，大幅提升科技成果转化率。

　　加强人才队伍建设。实施高端人才培养计划，以高层次人才和高技能人才为重点，深化“人才特区”建设，实施好“黄河三角洲学者”、“金蓝领”培训等重大人才工程；落实人才政策，吸引一批高层次海外人才、创新型科技人才和产业领军人才来东营创业发展。加大知识产权保护和开发力度，健全人才服务体系，营造尊重人才、支持创业、鼓励创新的良好环境。

　　(七)着力保障改善民生，在和谐社会建设上取得新成效。人民对美好生活的向往，就是我们的奋斗目标。我们要不断加大民生投入，尽最大努力解决群众生产生活困难，让人民群众生活越来越幸福美好。

　　积极促进创业就业。(1)实行就业优先和更加积极的就业政策，推动实现更高质量的就业，全年新增城镇就业3.3万人，农村劳动力转移就业3.1万人。(2)统筹抓好重点群体就业，把促进以高校毕业生为重点的青年就业放在首位，规划建设大学生创业基地。加大就业援助力度，建立对就业困难人员和零就业家庭的动态帮扶机制。创建工友创业园。(3)办好新型农民学校，大规模培训农民。(4)深入开展全民创业活动，激发民间创业热情，以创业带就业，以创业兴家业。(5)加快公共就业服务平台建设，加强职业培训，不断完善就业服务体系。(6)加强劳动争议调解仲裁和劳动监察，积极构建和谐劳动关系。

　　不断完善城乡社会保障体系。(1)建立健全社会保障待遇确定机制和正常调整机制，适时提高基本养老金、失业保险金等标准。(2)抓好城乡居民医疗保险整合工作，城乡居民医保财政补助标准提高到每人每年320元，市级城乡居民大额医药费财政补助标准提高到1000万元。(3)提高社会救助水平，城市低保标准每人每月提高30元，农村低保标准每人每年提高340元，农村五保供养标准每人每年提高500元，将老年人救助范围扩大到75岁及以上城镇非离退休老年人。(4)鼓励发展社会福利、慈善和社会养老服务事业，提高残疾人社会保障和服务水平，规划建设市级养老服务中心，实施残疾人康复救助工程。(5)实施保障房建设和棚户区改造工程，年内新建保障性住房6631套；支持胜利油田、石油大学、济军生产基地职工住房建设。

　　大力发展社会事业。一是坚持教育优先发展。进一步加大教育投入，推进教育现代化建设。全面实施素质教育，着力提高教育质量。(1)全面完成中小学办学条件标准化、校舍安全和农村中小学“两热一暖一改”工程，继续抓好幼儿园标准化建设。(2)加快实施金融小学、胜利五中、特校和实验学校建设改造工程，新建东城5所学校、幼儿园，统筹规划建设市中等专业学校、市体校、市二中、市一中。(3)实施学校现代化建设工程，建立市级教育信息资源公共服务平台和现代化学校项目库。(4)积极探索学生用车、营养餐等管理运行机制，抓好农村义务教育学校营养厨房建设。(5)支持东营职业学院建设国家骨干高职院校，加快市技师学院二期工程建设。积极引进高等教育资源。(6)加强师德建设，提高教师队伍素质。二是加快发展医药卫生事业。深化医药卫生体制改革，努力提高人民健康水平。(1)积极推进公立医院改革，全面实行药品零差价销售，创新体制机制，推行便民惠民措施。(2)巩固完善基本药物制度，深化基层医疗卫生机构综合改革，加强以全科医生为重点的基层人才队伍建设。(3)支持市人民医院创建“三甲”医院，抓好市人民医院急诊急救中心、市疾病预防控制中心建设。三是做好人口计生工作。(1)夯实基层基础，稳定低生育水平，努力提高人口素质。(2)积极开展计划生育“幸福家庭”创建活动。扎实开展双拥共建，做好国防教育、人民防空、民兵预备役等工作。关心支持妇女儿童、青少年和老龄事业发展。进一步提高外事侨务、民族宗教、无线电管理工作水平。积极推进档案、仲裁、气象、对台、史志、红十字会、防震减灾等工作。继续做好对口支援工作。

　　推动文化繁荣发展。以建设文化强市为目标，推动文化事业全面繁荣、文化产业快速发展。加快大剧院、奥体中心、群众文化艺术馆、黄河文化博物馆、图书馆、方志馆等重点场馆建设，深入实施文化惠民工程，积极创建全省公共文化服务体系示范区。深入挖掘优秀传统文化，办好孙子国际文化旅游节等活动，打造黄河口特色文化品牌。做好第十届中国艺术节筹备工作。改造提升传统文化产业，积极发展文化创意、动漫游戏等新兴文化产业，培育一批文化企业、产业项目和产业园区。办好黄河口国际马拉松赛，广泛开展全民健身运动，促进群众体育和竞技体育全面发展。发展哲学社会科学、新闻出版、广播影视、文学艺术事业。深入推进全国文明城市创建工作，广泛开展群众性精神文明创建活动，提高全社会文明程度。大力推进依法治市，增强全社会学法尊法守法用法意识，提高民主法制建设水平。

　　加强和创新社会管理。构建社会管理综合治理新机制，实施6项社会管理创新示范项目。夯实社会管理基层基础，健全基层服务网络。坚持以群众工作统领信访工作，健全群众利益诉求表达机制和社会矛盾调解机制，有效化解各类不稳定因素。深入推进“平安东营”建设，完善社会治安和油区治安打防控一体化布局，严厉打击各类违法犯罪活动。扎实推进国家安全发展示范城市创建工作，夯实安全生产基层基础，加强重点行业、领域监管，坚决防止发生重特大安全事故。高度重视食品药品安全，加强生产、流通各重点环节监管，严肃查处食品药品安全事件。健全应急管理体制，有效预防和处置各类突发公共事件。

　　(八)着力加强政府自身建设，在提高行政效能上取得新成效。形势的发展、事业的开拓、人民的期待，都要求我们以改革创新的精神全面加强政府自身建设，切实担当起实现“两个率先”的重任。

　　要推进依法行政，建设法治政府。着力完善科学民主决策程序，健全重大事项集体决策、专家咨询、社会公示与听证等制度。扎实开展行政复议委员会试点工作。规范执法行为，加强执法监督，做到严格规范公正文明执法。大力推行政务公开，抓好政府门户网站、市长公开电话、政务微博、行风热线等平台建设，提高政府工作透明度。自觉接受人大法律监督、工作监督和政协民主监督，高度重视新闻舆论和社会公众监督，广泛听取社会各界的意见和建议。

　　要切实改进作风，建设服务政府。认真落实中央关于改进工作作风、密切联系群众八项规定，大兴求真务实之风，做到察实情、办实事、求实效。牢固树立正确的政绩观，不讲不实之词，不干不实之事，不贪一时之功，多干打基础、利长远的工作，多办解民忧、惠民生的实事。强化群众观点，真诚倾听群众呼声，真实反映群众愿望，真情关心群众疾苦。认真开展群众路线教育实践活动。大力弘扬勇于担当精神，大力推进思想解放，心无旁骛干工作，聚精会神促发展，把全部心思和精力倾注到推进“两个率先”上来，把所有智慧和力量凝聚到干事创业中来，努力争创一流业绩。

　　要强化管理创新，建设效能政府。优化政府组织结构，加快构建权责明晰、分工合理、执行有力的行政管理体制。深化行政审批制度改革，推行网上审批，提高行政审批效率。严格执行政府工作规则，规范办事程序，推动政府工作规范有序运转。完善目标责任制，改进督查考核方式，加大重点工作督查力度，强势强力推进工作落实。加强行政效能监察，严格行政问责，确保政令畅通、令行禁止。

　　要坚持从严治政，建设清廉政府。认真落实党风廉政建设责任制，全面推进惩治和预防腐败体系建设，着力健全长效机制。加强反腐败工作，突出工程建设、政府采购、资金使用等重点领域和重要环节，健全完善制度，深化专项治理，努力从源头上预防和治理腐败。坚决纠正损害群众利益的不正之风，认真解决群众反映强烈的突出问题。坚决查处各类违纪违法案件，严厉惩治腐败分子。严控“三公”经费支出，降低行政成本。全体政府工作人员要牢固树立廉洁从政意识，提高拒腐防变能力，以为民务实清廉的实际行动树立形象、取信于民。

各位代表，担当成就伟业，拼搏创造辉煌。让我们紧密团结在以习近平同志为总书记的党中央周围，高举中国特色社会主义伟大旗帜，以邓小平理论、“三个代表”重要思想、科学发展观为指导，在省委、省政府和市委的坚强领导下，全力实施黄蓝国家战略，解放思想，开拓创新，凝聚力量，攻坚克难，为夺取全面建成小康社会新胜利、谱写生态文明典范城市建设新篇章而努力奋斗！
